# Topiramate alters the gut microbiome to aid in its anti-seizure effect

**DOI:** 10.3389/fmicb.2023.1242856

**Published:** 2023-10-10

**Authors:** K'Ehleyr Thai, Michael W. Taylor, Tatiane Fernandes, Eunice A. Akinade, Susan L. Campbell

**Affiliations:** ^1^Graduate Program in Translational Biology Medicine and Health, Virginia Tech, Roanoke, VA, United States; ^2^School of Animal Sciences, Virginia Polytechnic Institute and State University, Blacksburg, VA, United States; ^3^School of Neuroscience, Virginia Polytechnic Institute and State University, Blacksburg, VA, United States

**Keywords:** *Lactobacillus johnsonii*, anti-epileptic drugs, anti-seizure medication, PTZ, butyrate, epilepsy, gut microbiome, gut microbiota

## Abstract

**Introduction:**

There is a growing interest in the role of the gut microbiota in epilepsy, however, it is unclear if anti-seizure medications (ASMs) play a role in the gut-brain axis. To test this, we investigated the impact of the ASM topiramate on the gut microbiome of mice.

**Methods:**

C57BL/6J mice were administered topiramate in their drinking water for 5 weeks. 16S ribosomal RNA gene sequencing was performed on fecal samples collected at 5 weeks. Analysis of alpha diversity, beta diversity, and differential abundance were performed. Cecal contents were analyzed for short-chain fatty acids (SCFAs) composition. Pentylenetetrazol (PTZ)-kindling was performed in saline, topiramate, *Lactobacillus johnsonii*, and topiramate and *Lactobacillus johnsonii* treated mice. Mice received PTZ injection every other day for a total of twelve injections, seizure activity was video monitored for 30 minutes and scored.

**Results and discussion:**

Our study revealed that topiramate ingestion significantly increased *Lactobacillus johnsonii* in the gut microbiome of naïve mice. Treatment with topiramate and *Lactobacillus johnsonii* together, but not alone, reduced susceptibility to PTZ-induced seizures. Co-treatment also significantly increased the percent of butyrate and the abundance of butyrate-producing family *Lachnospiraceae* in the gut, and elevated the GABA/glutamate ratio in the cortex. Our results demonstrate that an ASM can alter the gut microbiome to aid in their anti-seizure effect *in vivo* and suggest the potential of the probiotic *Lactobacillus johnsonii* as an adjunct therapy with topiramate in reducing seizure susceptibility.

## 1. Introduction

Epilepsy remains one of the most common neurological disorders worldwide, with a global impact of 65 million people (Beghi, 2020; Kanner and Bicchi, 2022). It is characterized by spontaneous recurrent seizures, and since there is no cure, the primary focus in treating epilepsy is to reduce seizures, often via anti-seizure medications (ASMs). When two tolerated and appropriately prescribed ASMs fail to achieve seizure freedom, the patient is deemed drug-resistant. Currently, there are over 30 major ASMs approved for use in the United States, yet about one-third of the epileptic population is drug-resistant, a number that remains unchanged despite the increasing number of ASMs that have come to market (Chen et al., 2018). Thus, patients need alternative therapeutic strategies to treat seizures. The gut-brain axis has received increasing attention as alterations in the gut microbiome and specific compositional changes can reduce seizure frequency in humans and rodents (Ghanizadeh and Berk, 2015; He et al., 2017; Braakman and van Ingen, 2018; Gómez-Eguílaz et al., 2018; Olson et al., 2018). While most studies are aimed at understanding how changes in the gut microbiome impact seizures, there has been little investigation into the potential impact of ASMs on the gut microbiota and whether it may play a role in the anti-seizure effect of ASMs.

Current studies have focused on the effects of ASMs on microbial growth in culture (Esiobu and Hoosein, 2003; Ilhan et al., 2022). These studies have shown the potential for ASMs, such as sodium valproate and lamotrigine, to have antimicrobial properties (Qian et al., 2009; Stokes et al., 2014). In addition, a few studies have also shown that the gut microbiota can metabolize ASMs in rodents and that ASMs may alter the gut microbiome of rodents and humans (Elmer and Remmel, 1984; Kitamura et al., 1997; Cussotto et al., 2019; Gong et al., 2022). These studies show the potential for ASMs to interact with the gut microbiota; however, it is unknown whether this may impact their anti-seizure effect.

Topiramate [2,3:4,5-Bis-O-(1-methylethylidene)-β-D-fructopyranose sulfamate] is a second-generation ASM prescribed to treat both partial and generalized seizures and migraines (Fariba and Saadabadi, 2023). Several mechanisms have been proposed in its anti-seizure effect, including enhancement of γ-aminobutyric acid type A (GABA_A_) receptor activity, blockade of voltage-gated Na^+^ channels, reduction of membrane depolarization via α-amino-3-hydroxy-5-methyl-4-isoxazolepropionic acid (AMPA)/Kainate receptors, and weak carbonic anhydrase inhibitory activity. In addition, topiramate is a unique ASM as it is a sulfamate-substituted monosaccharide, which is an uncommon structure for ASMs (Maryanoff et al., 1998). Finally, topiramate is a well-established ASM with efficacy as a monotherapy in generalized seizures and as an add-on treatment in drug-resistant focal epilepsy (Bai et al., 2022b).

In this study, we investigated the effect of topiramate on the composition of the gut microbiome. Our data revealed that topiramate alters the gut microbiome of naïve mice by significantly increasing *Lactobacillus johnsonii*. Using a model of pentylenetetrazol (PTZ)-kindling, we found that *Lactobacillus johnsonii* aids topiramate in reducing seizure susceptibility in mice. Finally, the co-treatment of topiramate with *Lactobacillus johnsonii* increases the relative abundance of butyric acid and *Lachnospiraceae*, a butyrate-producing family, as well as increases the GABA/Glutamate ratio in the cortex, both of which may contribute to *Lactobacillus johnsonii's* anti-seizure effect. To the best of our knowledge, this is the first study to show that ASMs may impact the gut microbiota, which in turn impacts the efficacy of ASMs.

## 2. Materials and methods

### 2.1. Animals

Animals were housed and handled according to the guidelines of the National Institutes of Health Committee on Laboratory Animal Resources. Prior approval of the Virginia Polytechnic Institute and State University Institutional Animal Care and Use Committee was obtained for all experimental protocols. C57BL/6J male mice aged 7–8 weeks were purchased from Jackson Laboratory. Mice were allowed to acclimate for 7 days prior to the start of experiments and were provided sterile water and irradiated chow (Teklad 2918) *ad libitum*. All animals were handled using a sterile technique and housed in a facility with ambient temperature providing a 12-h light/dark cycle. All efforts were made to minimize animal pain and the number of animals.

### 2.2. Topiramate treatment and fecal collection

Topiramate (Sigma Aldrich 1672206) (80 mg/kg) was dissolved into the drinking water of mice and given *ad libitum* for 5 weeks. Water was changed every 2–3 days. Once a week, mice were weighed, and fecal samples were collected in DNA-free Eppendorf tubes (Eppendorf 022600028). Fecal samples were immediately put on dry ice and stored at−80°C until processing. Mice were housed two per cage to reduce the cage effect.

### 2.3. 16s rRNA sequencing and analysis

DNA was isolated from fecal samples using the ZymoBIOMICS DNA Miniprep Kit (Zymo D4300) following the manufacturer's protocol. 16S rRNA sequencing analysis was performed as previously described (Gallucci et al., 2021). The universal primers 515F and 926R were used to amplify the V4-V5 region of the 16S rRNA gene per the Earth Microbiome Project protocol (https://www.earthmicrobiome.org/). The V4-V5 regions were sequenced using the MiSeq v3 600-cycle kit on the MiSeq platform (Illumina), resulting in 2 × 300 paired-end sequences.

The raw sequences were processed and analyzed with QIIME2 v2020.2 (Bolyen et al., 2019). Forward and reverse reads were quality filtered, trimmed, and joined with DADA2, as well as used to denoise joined reads to amplicon sequence variants (ASVs) (via q2-dada2) (Callahan et al., 2016). ASVs were aligned to a phylogenetic tree using an insertion method to the SILVA 128 SEPP reference database (via q2-fragment-insertion-sepp) (Janssen et al., 2018). Alpha and beta diversity metrics were calculated using samples rarified to 45,881 and 26,986 sequences per sample, respectively (via q2-diversity). Features were filtered if they did not pass the requirements of appearing in a minimum of two samples, or 10% of the total sample number, and a minimum frequency level of 0.1% of the mean frequency per sample due to the reported Illumina MiSeq bleed-through between runs; a pseudo-count of 1 was added. Filtered feature tables at the genus level were used in the linear discriminant effect size analysis (LEfSe), which was performed using standard parameters with Kruskal-Wallis (*p* < 0.05) to obtain significant LDA scores (LDA > 2) via the Galaxy browser (Segata et al., 2011; Gallucci et al., 2021; The Galaxy Community, 2022). Filtered feature tables at the family level were used to measure differential abundance by ANCOM (via q2-composition ANCOM) using default parameters to obtain significant W scores (Mandal et al., 2015).

### 2.4. SCFA analysis

Thawed samples were weighed and diluted in distilled water in a ratio of 1 g of cecal content to 2 ml of distilled water, vortexed for 3 min at maximum speed using the Vortex Genie 2, and allowed to rest overnight at 4°C. The samples were then centrifuged at 1,000 × *g* for 5 min, and the supernatant was collected. Next, 0.1 ml of the supernatant was acidified with 0.17 ml of metaphosphoric acid (25%, w/v), and 0.13 ml of internal standard (5 mmol, 4-methyl-valeric acid, Sigma, St. Louis, MO, United States) was added and vortexed, and the solution was allowed to rest for 30 min at 4°C. The samples were then centrifuged at 3,000 × *g* for 15 min. The supernatant was collected and used for short-chain fatty acid (SCFA) determination using a 6890 N Network GC System gas chromatograph (Agilent Technologies) equipped with a flame ionization detector as previously described (Izuddin et al., 2019). One microliter of the sample was injected at split 1:30 at a temperature of 230°C. Separation of the SCFA profile was determined using a Quadrex 007-10 Series (Quadrex Corp., New Haven, CT 06525, United States) bonded-phase fused silica capillary column (15 m, 0.250-mm internal diameter, and 0.25-μm film thickness). The temperature of the column was set at 60°C held for 2 min, increased to 100°C (10°C/min), increased to 200°C (20°C/min), and held for 5 min. Nitrogen gas was supplied as a carrier gas at 1 ml/min. The temperature of the detector was set at 230°C. Commercial standards (Sigma-Aldrich, St. Louis, MO, United States) of acetic (45,997), propionic (94,425), iso-butyric (46,935), butyric (19,215), iso-valeric (78,651), valeric (75,054), and caproic (21,529) acids were used as external standards for peak identification. The molar concentration of SCFA was identified based on a single point of the internal standard and a calibration curve with external standards.

### 2.5. *Lactobacillus johnsonii* isolation, colony PCR, and qPCR

One fecal pellet from a C57BL/6J mouse was vortexed in 1 ml of sterile 1 × PBS for 15 min at maximum speed. Next, the sample was centrifuged for 1 min at 500 × *g*. Glycerol was added to the supernatant to make a 20% solution and stored at−80°C. This solution was subcultured overnight in MRS broth at 37°C. The sub-culture was plated on MRS agar and allowed to grow overnight at 37°C. Colonies were chosen at random for colony PCR, which were split to continue growth in MRS broth and for colony PCR. This process was repeated until one culture had six randomly selected colonies positive for *Lactobacillus johnsonii*. This solution was then grown in MRS broth overnight at 37°C, centrifuged for 1 min at 500 × *g*, and glycerol was added to make a 20% glycerol solution.

qPCR was performed using the DNA extracted from the fecal pellets as described above. qPCR was performed using the Biorad SsoAdvanced Universal SYBR Green Supermix. A volume of 8 μl of DNA at 50 ng was used in a 20 μl reaction consisting of 1 μl of forward primer at 10 μM, 1 μl of reverse primer at 10 μM, and 10 μl of SYBR Green Master Mix. Reactions were performed using the Biorad CFX96 Touch Real-time PCR Detection System. The reaction was run at 98°C for 3 min, followed by 49 cycles at 98°C for 10 s and 60°C for 40 s for plate reading: *Lactobacillus johnsonii* F: AGAGAGAAACTCAACTTGAAATA R: CCTTCATTAACCTTAACAGTTAA, *Lactobacillus gasseri* F: TCAAGAGCTGTTAAGGCTGT R: CTATCGCTTCAAGTGCTTTC, and total bacteria F: ACTCCTACGGGAGGCAGCAGT R: ATTACCGCGGCTGCTGGT.

### 2.6. PTZ kindling and seizure scoring

C57BL/6J mice aged 9 weeks were administered sub-convulsive doses of PTZ (Sigma Aldrich P6500) (35 mg/kg) i.p. every other day for a total of 12 injections. PTZ was freshly dissolved in saline (0.9% NaCl). Mice were video-monitored and scored for 30 min following each injection. Seizure scores were determined based on a modified Racine scale as follows: 0 = no change; 1 = immobilization; 2 = head nodding, partial myoclonus; 3 = continuous whole-body myoclonus, whole-body jerks; 4 = rearing and falling, tonic seizure; 5 = tonic-clonic seizure with wild rushing and jumping; and 6 = death (Shimada and Yamagata, 2018).

Prior to the start of PTZ injections, mice that were to receive *Lactobacillus johnsonii* (10^9^ CFUs) as a treatment, either alone or in combination with topiramate, were given a once-daily oral gavage (Braintree Scientific N-PK 010) for 7 days; all others received saline (0.9% NaCl). Mice were divided into four groups with seven mice per group: saline (0.9% NaCl), *Lactobacillus johnsonii* (10^9^ CFUs), topiramate (50 mg/kg), and *Lactobacillus johnsonii* (2.5 × 10^9^ CFUs) + topiramate (50 mg/kg). Mice received an oral gavage of their respective treatments 1 h prior to PTZ administration on injection days.

Topiramate was freshly dissolved in saline (0.9% NaCl). *Lactobacillus johnsonii* was subcultured in MRS broth at 37°C every morning from an overnight culture at a 1:100 ratio. The subculture was centrifuged after 4–5 h of growth, at which the bacteria were in the exponential phase. A volume of 200 μl of the subculture was added to a 96-well plate to read the optical density at 600 nm on a microplate reader. CFUs were determined using a previously established standard curve.

### 2.7. Tissue processing and assays

The cortex was dissected and stored at −80°C until processing. Samples were processed in 750 μl of sucrose buffer and homogenized with Dounce glass homogenizers. The sucrose buffer contained 250 mM sucrose, 50 mM Tris pH 7.5, 25 mM KCl, and 10% protease and phosphatase inhibitors freshly added. After homogenization, samples were centrifuged at 7,700 × *g* for 1 min, and the supernatant was collected and used for GABA and glutamate assays. Protein measurements were read using a Bradford assay (Bio-Rad).

In total, 25 μg of protein per sample was used for each assay. GABA was measured using the LS Bio Mouse GABA Competitive ELISA kit (LS-F32284) following the manufacturer's instructions. Glutamate was measured using ThermoFisher Scientific's Amplex Red Glutamic Acid/Glutamate Oxidase assay kit (A12221), following the manufacturer's instructions.

### 2.8. Statistics

All statistics were conducted in GraphPad Prism 9 and 10 and reported as mean ± SD unless otherwise specified. The statistical tests used are reported with figures.

## 3. Results

### 3.1. Topiramate alters the beta diversity of the gut microbiome in naïve mice

**Figure 1:**
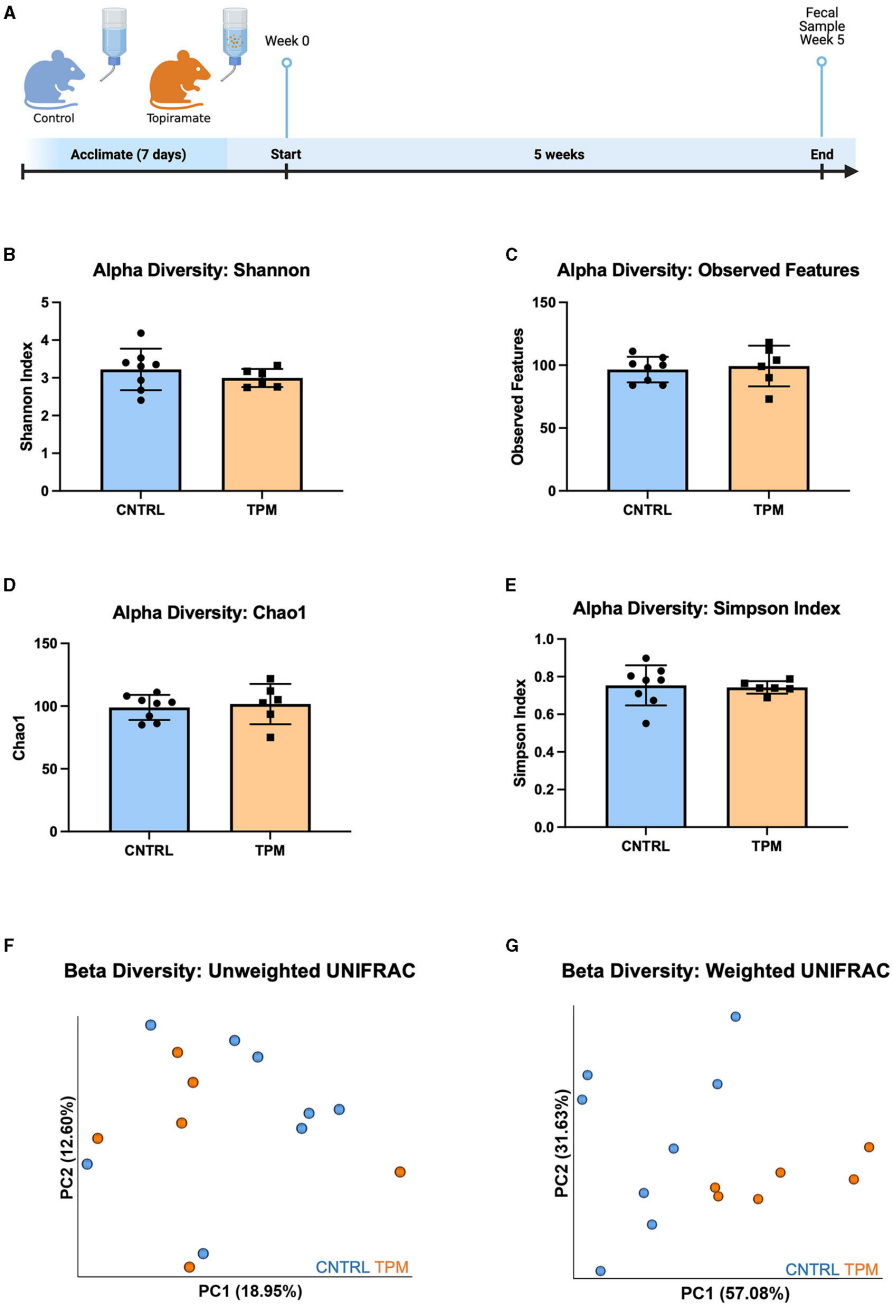
Alpha and beta diversity metrics comparing the gut microbiome of mice treated with topiramate or controls. **(A)** Experimental timeline (Adapted from “Mouse Experimental Timeline”, by BioRender.com (2023). Retrieved from https://app.biorender.com/biorender-templates). Alpha diversity metrics using the **(B)** Shannon index, **(C)** observed features, **(D)** Chao1, and **(E)** Simpson Index. Beta diversity is measured by the **(F)** unweighted UNIFRAC and **(G)** weighted UNIFRAC statistical tests visualized by the principal coordinates (PC1 and PC2) analysis (PCoA). Each dot represents a fecal sample from an individual mouse; mice in the control group are blue, and mice in the topiramate-treated group are orange. Data are shown as mean ± SD. CNTRL = 8, TPM = 6.

To evaluate the effect of topiramate on microbial composition, naïve C57BL/6J male mice (9 weeks old) were treated with topiramate (80 mg/kg) or regular drinking water for 5 weeks (Figure 1A). Weight was monitored weekly, and there were no differences between control and topiramate-treated mice after 5 weeks (Supplementary Data 1). After 5 weeks of treatment fecal samples were compared between control and topiramate-treated mice using QIIME2 to analyze the V4 and V5 regions of the 16S rRNA gene. Alpha diversity was measured with the Shannon index, observed features, Chao1, and Simpson Index. Beta diversity was measured using weighted and unweighted UNIFRAC statistical tests. Analyses of alpha diversity and beta diversity using an unweighted UNIFRAC statistical test were not altered due to topiramate treatment (*p* = 0.3450; Figure 1B, *p* = 0.5481; Figure 1C, *p* = 0.7079; Figure 1D, *p* = 0.8128; Figure 1E, *p* = 0.328; Figure 1F). However, topiramate treatment altered the beta diversity using a weighted UNIFRAC statistical test (*p* = 0.003, 999 permutations, PERMANOVA; Figure 1G).

### 3.2. Relative abundance of *Lactobacillus johnsonii* is increased in topiramate-treated mice

**Figure 2:**
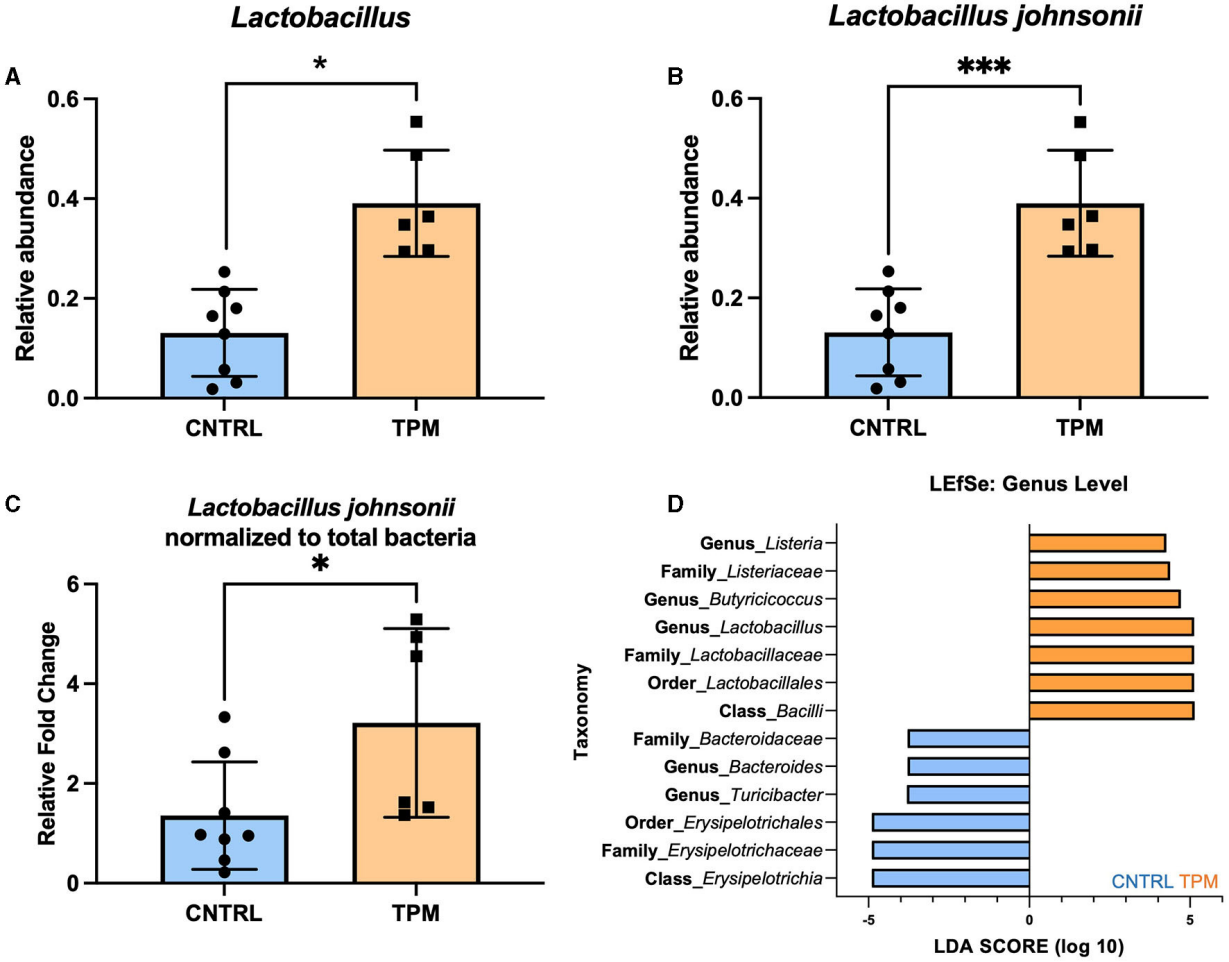
Topiramate increases *Lactobacillus johnsonii* in the gut microbiome of naïve mice. **(A)** Relative abundance plot of the genus *Lactobacillus* in the gut microbiome of naïve mice after 5 weeks of topiramate treatment. Further analysis revealed that at the species-level **(B)** *Lactobacillus johnsonii* is significantly increased due to topiramate treatment. **(C)** qPCR with validated primers targeting *Lactobacillus johnsonii* and total bacteria. **(D)** Linear discriminant analysis (LDA) in addition to effect size (LEfSe) also shows *Lactobacillus, Butyricocccus*, and *Listeria* as defining features in topiramate-treated mice. LEfSe identifies *Bacteroides* and *Erysipelotrichaceae* as defining features of control mice. Bars in orange are topiramate features, and bars in blue are control features. Data are shown as mean ± SD. *p < 0.05, ***p < 0.001.

The significance of the weighted UNIFRAC but not the unweighted UNIFRAC test suggested that there may not be differing bacteria between the two groups but an alteration in the abundance of specific microbes. Analysis of the relative abundance at the genus level revealed that topiramate treatment significantly increased the abundance of *Lactobacillus* (*p* = 0.045971, multiple unpaired *t*-tests with Welch correction; Figure 2A). Next, we sought to determine if there were any dominant species driving this change. Analysis of the four reported *Lactobacillus* species showed that *Lactobacillus johnsonii* was significantly different between the control and topiramate-treated groups (*p* = 0.001196, multiple unpaired *t*-tests with Welch correction; Figure 2B). Phylogenetic mapping using a SILVA database listed this bacterium as *Lactobacillus gasseri* but was confirmed to be *Lactobacillus johnsonii* via PCR (Supplementary Data 2). This was further established via qPCR and linear discriminant analysis (LDA) effect size (LEfSe). Using validated primers, DNA extracted from fecal samples showed an increase in the relative fold change of *Lactobacillus johnsonii* normalized to total bacteria in topiramate-treated mice (*p* = 0.0375, unpaired *t*-test; Figure 2C). In addition, LEfSe identified the genus *Lactobacillus* and genus *Butyricicoccus* in the topiramate-treated group and genus *Bacteroides* and *Turicibacter* and class *Erysipelotrichia* in the controls (Figure 2D).

Cecal content of control and topiramate-treated mice was analyzed for SCFA composition. No differences in the amount of SCFA were observed between the control and topiramate-treated mice (Supplementary Data 3). However, a simple linear regression revealed a correlation between the concentration of butyric acid, propionic acid, and acetic acid and the relative abundance of *Lactobacillus johnsonii* in the control and topiramate-treated mice (Supplementary Data 4). In control mice, these SCFAs had a negative or no correlation to the abundance of *Lactobacillus johnsonii* (*p* = 0.4886; Supplementary Figure S4D, *p* = 0.6832; Supplementary Figure S4E, *p* = 0.0292; Supplementary Figure S4F). However, in topiramate-treated mice, these SCFAs were positively correlated or showed a positive trend with the abundance of *Lactobacillus johnsonii* (*p* = 0.0364; Supplementary Figure S4A, *p* = 0.0006; Supplementary Figure S4B, *p* = 0.1383; Supplementary Figure S4C). This suggests that the positive correlation seen only in the topiramate-treated mice between major SCFAs and *Lactobacillus johnsonii* may be due to either the high abundance of *Lactobacillus johnsonii* in the topiramate-treated group, the ingestion of topiramate, or an interaction between topiramate and *Lactobacillus johnsonii*.

### 3.3. *Lactobacillus johnsonii* aids topiramate in reducing PTZ-induced seizure susceptibility

**Figure 3:**
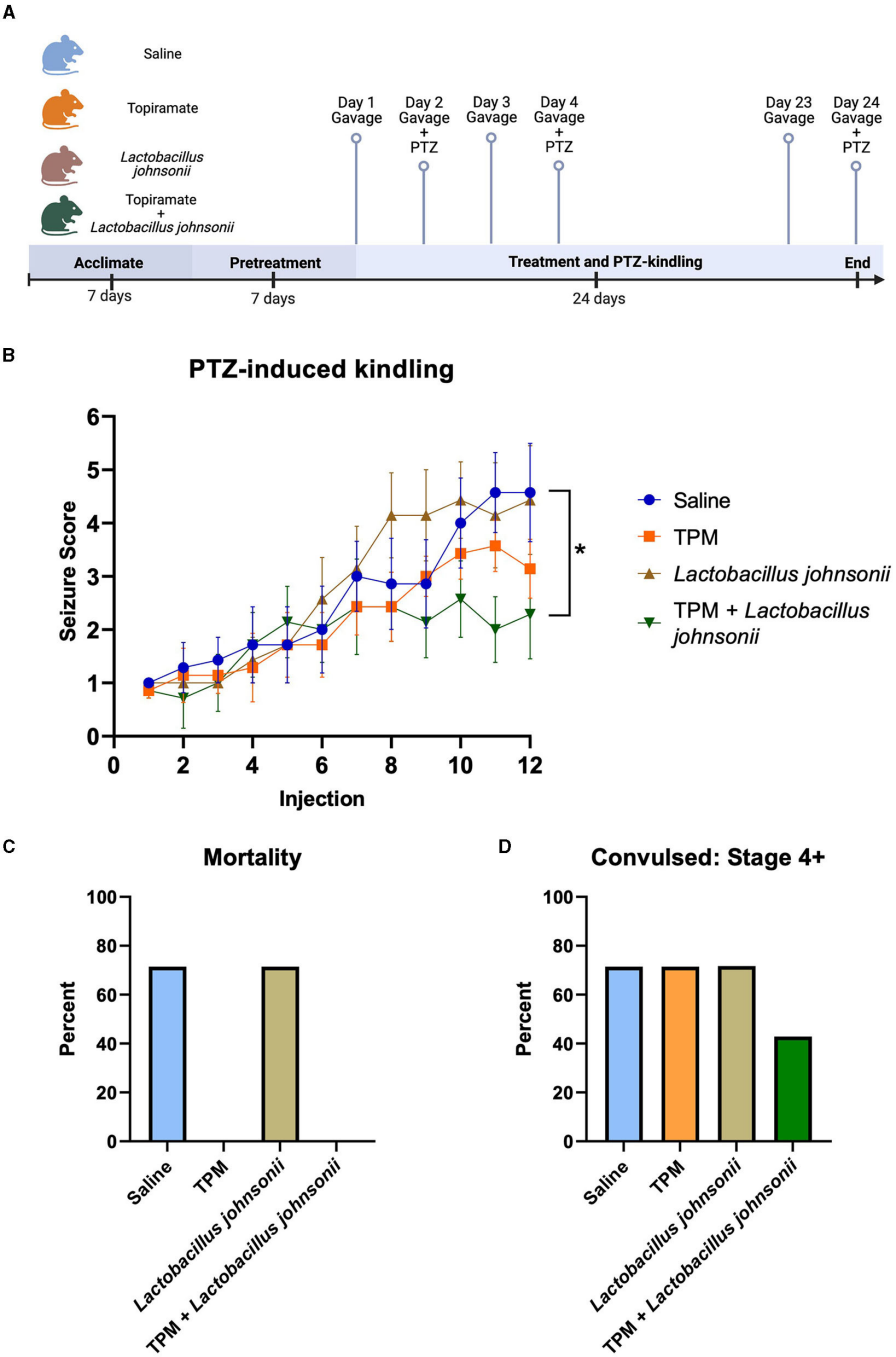
Co-treatment with topiramate and *Lactobacillus johnsonii* significantly reduces seizure susceptibility in PTZ kindling. **(A)** Experiment timeline. Adapted from “Mouse Experimental Timeline”, by BioRender.com (2023). Retrieved from https://app.biorender.com/biorender-templates. **(B)** Summary data of PTZ-induced seizure scores in saline, topiramate, *Lactobacillus johnsonii*, and topiramate and *Lactobacillus johnsonii*-treated mice. **(C)** Percent mortality in response to PTZ. **(D)** Data of the percent of mice that exhibited a stage 4 or above seizure score. Seizure activity was measured by scoring seizure behaviors corresponding to the modified Racine scale. Data are shown as mean ± SEM. *p < 0.05. *N* = 7 per group.

Since topiramate increased the relative abundance of *Lactobacillus johnsonii*, we sought to determine if *Lactobacillus johnsonii* impacted topiramate's anti-seizure effect. C57BL/6J mice (9 weeks old) received a daily oral gavage of either saline, topiramate (50 mg/kg), *Lactobacillus johnsonii*, or a combination of topiramate (50 mg/kg) and *Lactobacillus johnsonii*. Mice receiving *Lactobacillus johnsonii* were pre-treated for 7 days with 10^7^ CFUs, and all others received saline (Figure 3A). After 7 days, all mice received a sub-convulsive dose of PTZ every other day, 1 h after the gavage, for a total of 12 injections. All mice were video-monitored for 30 min after PTZ injection, and seizure activity was scored using a modified Racine scale. We found that after 12 injections, mice treated with both topiramate and *Lactobacillus johnsonii* had significantly reduced seizure susceptibility compared to saline controls (*p* = 0.0339, Friedman test with Dunn's correction for multiple comparisons; Figure 3B). Treatment with topiramate (*p* = 0.1062) or *Lactobacillus johnsonii* (*p* > 0.9999) alone did not significantly impact seizure scores compared to saline controls; however, the use of topiramate was protective against mortality (Figure 3C). Moreover, co-treatment of topiramate and *Lactobacillus johnsonii* reduced the number of mice that exhibited Stage 4 or greater convulsive seizures (Figure 3D).

### 3.4. Topiramate and *Lactobacillus johnsonii* together remodel the gut microbiome in response to PTZ-induced seizures

**Figure 4:**
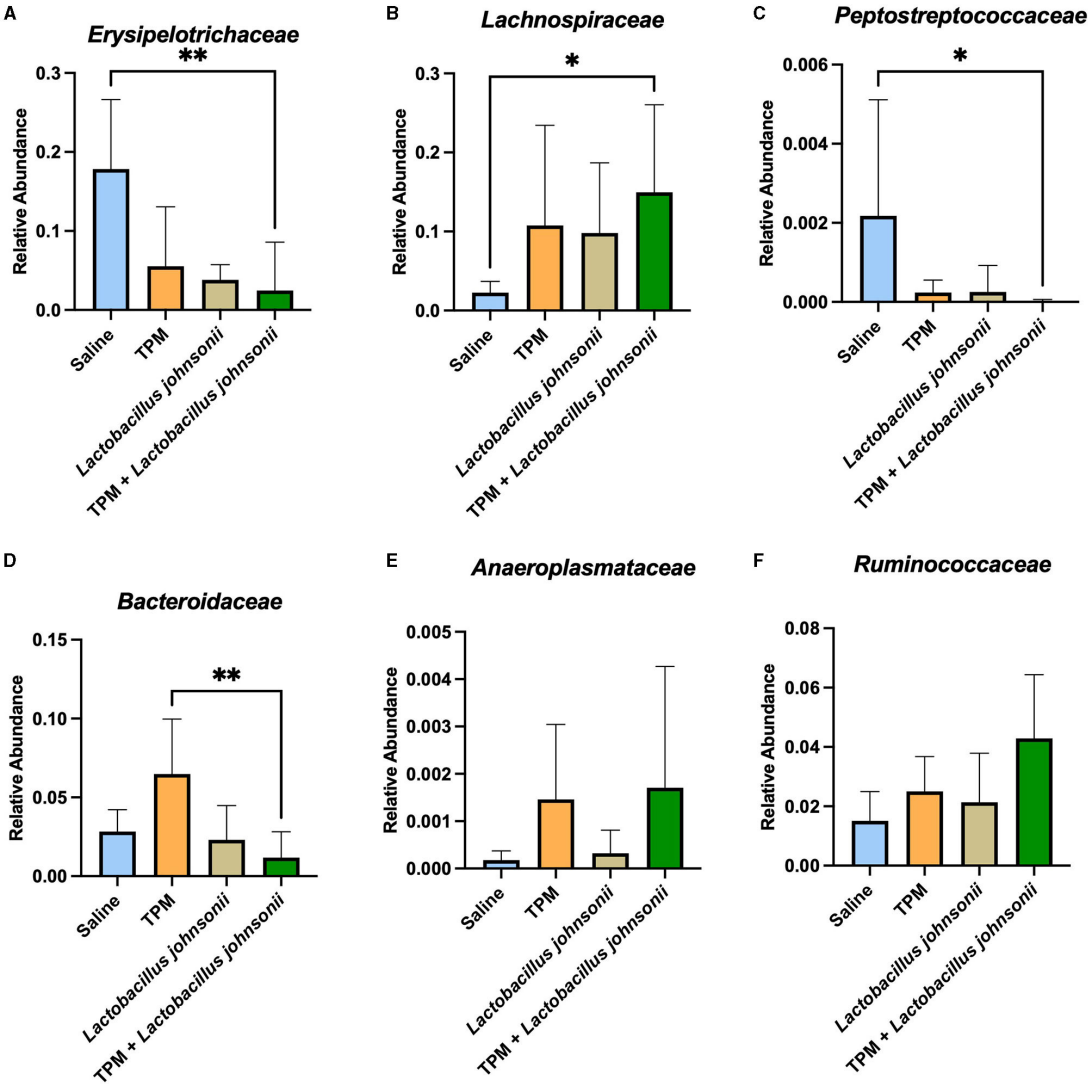
Remodeling of the gut microbiome composition by topiramate and *Lactobacillus johnsonii* co-treatment after PTZ kindling. ANCOM identified **(A–E)** five families that were significantly different across the groups. Kruskal-Wallis with Dunn's multiple comparisons was performed on the five families identified. **(A, C)** *Erysipelotrichaceae* and *Peptostreptococcaceae* are significantly reduced in the co-treatment group compared to saline treatment. **(B)** *Lachnospiraceae* is significantly increased in the co-treatment group when compared to saline treatment. **(D)** *Bacteroidaceae* is significantly decreased in the co-treatment group compared to the topiramate-treated group. **(E)** Although identified by ANCOM, there were no significant differences between the groups in *Anaeroplasmataceae* when using further statistical tests. **(F)** Not identified by ANCOM, the butyrate-producing family, *Ruminococcaceae*, trends toward an increase in the co-treatment group. Data are shown as mean ± SD. *p < 0.05, **p < 0.01. Saline = 6, TPM = 7, *Lactobacillus johnsonii* = 7, TPM + *Lactobacillus johnsonii* = 7.

As our data showed that topiramate and *Lactobacillus johnsonii* co-treatment conferred protection against PTZ-induced seizures, we analyzed the fecal samples of mice that underwent PTZ-kindling with and without treatment to determine if there were changes in microbial composition. We found no alteration in alpha diversity, Shannon index, observed features, Chao1, or Simpson index (Supplementary Figures S5A–D) among PTZ-treated mice that were administered saline, topiramate, *Lactobacillus johnsonii*, or topiramate and *Lactobacillus johnsonii*. However, there was a significant difference in beta diversity between unweighted UNIFRAC (*p* = 0.001, 999 permutations, PERMANOVA; Supplementary Figure S5E) and weighted UNIFRAC (*p* = 0.019, 999 permutations, PERMANOVA; Supplementary Figure S5F) among the groups. Differential abundance with ANCOM performed at the family level showed that *Erysipelotrichaceae* (W = 4), *Lachnospiraceae* (W = 3), *Peptostreptococcaceae* (W = 1), *Bacteroidaceae* (W = 1), and *Anaeroplasmataceae* (W = 1) were significantly different. A Kruskal-Wallis with Dunn's multiple comparisons was used on each family identified by ANCOM to further elucidate the differences between the groups. The relative abundance of *Erysipelotrichaceae* and *Peptostreptococcaceae* was significantly lower in the topiramate + *Lactobacillus johnsonii* group compared to saline (*p* = 0.0022; Figure 4A, *p* = 0.0426; Figure 4C). *Lachnospiraceae* was significantly increased in the topiramate + *Lactobacillus johnsonii* group compared to saline (*p* = 0.0116; Figure 4B). In addition, *Bacteroidaceae* was significantly reduced in the topiramate + *Lactobacillus johnsonii* group compared to the topiramate group (*p* = 0.0063; Figure 4D). Finally, there were no differences in *Anaeroplasmataceae* using a Kruskal-Wallis test (*p* = 0.3190; Figure 4E), despite ANCOM identifying it as differentially abundant. Interestingly, Figure 4F shows a trend toward an increase in the butyrate-producing family *Ruminococcaceae* in the topiramate and *Lactobacillus johnsonii*-treated group, although it was not identified by ANCOM.

### 3.5. Co-administration of topiramate and *Lactobacillus johnsonii* alters SCFA composition

**Figure 5:**
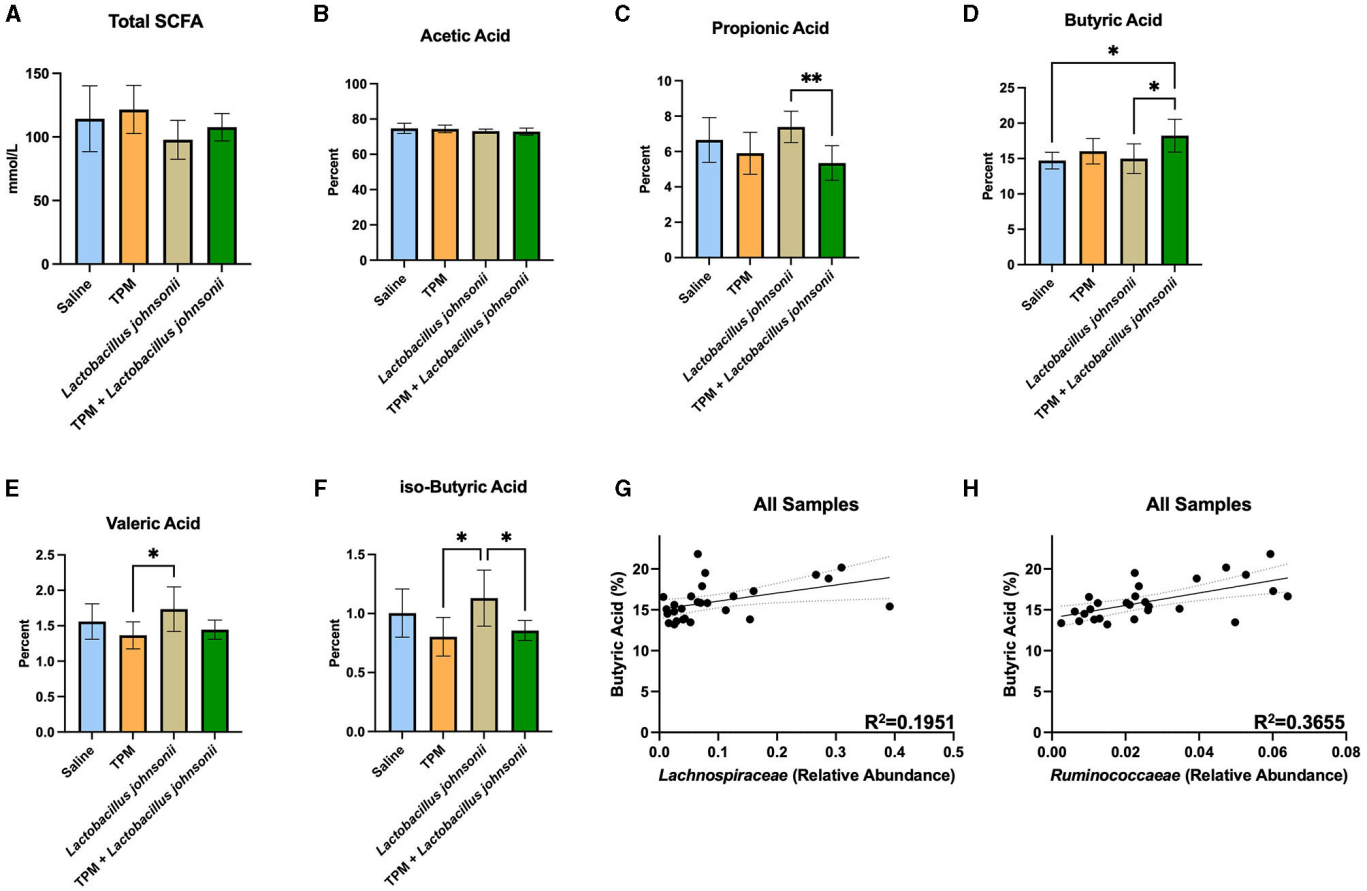
Alteration of SCFAs in response to co-treatment of topiramate and *Lactobacillus johnsonii*. **(A, B)** There were no significant changes in the total amount of SCFAs or the percentage of acetic acid in the ceca of mice after treatment and PTZ kindling. **(C)** Propionic acid was significantly reduced in the co-treatment groups compared to the *Lactobacillus johnsonii* treatment alone. **(D)** Butyric acid was significantly increased in the co-treatment group when compared to the saline or *Lactobacillus johnsonii* treatment alone. **(E)** Valeric acid was increased in the *Lactobacillus johnsonii* treatment when compared to the topiramate treatment. **(F)** Significant reductions in iso-butyric acid in the topiramate and the topiramate and *Lactobacillus johnsonii* group compared to the *Lactobacillus johnsonii* group alone. A simple linear regression showed a positive correlation among the abundance of butyrate-producing families **(G)** *Lachnospiraceae* and **(H)** *Ruminococcaceae* and the percentage of butyrate across all treatments. Data are shown as mean ± SD or regression lines with 95% confidence bands. *p < 0.05, **p < 0.01. Saline = 6, TPM = 7, *Lactobacillus johnsonii* = 7, TPM + *Lactobacillus johnsonii* = 7.

The ceca of mice that underwent PTZ-kindling were analyzed for SCFA composition (Figure 5; Supplementary Data 6). One-way ANOVAs with Tukey's multiple comparisons test with a single pooled variance between all groups were performed on all SCFAs (Figures 5A–F). The data revealed no significant difference in the total amount of SCFAs (*p* = 0.1201; Figure 5A). Analysis of the percent of SCFAs showed no significant differences in acetic acid (*p* = 0.3034; Figure 5B), caproic acid (data not shown), or iso-valeric acid (data not shown). However, propionic acid was reduced in the topiramate and *Lactobacillus johnsonii* treatment groups compared to *Lactobacillus johnsonii* treatment alone (*p* = 0.0090; Figure 5C). Butyric acid was increased (Figure 5D) in the topiramate and *Lactobacillus johnsonii* groups compared to saline (*p* = 0.0168) and *Lactobacillus johnsonii* treatment alone (*p* = 0.0225). *Lactobacillus johnsonii* treatment increased valeric acid compared to the topiramate treatment (*p* = 0.0317; Figure 5E) and increased iso-butyric acid (Figure 5F) compared to the topiramate treatment (*p* = 0.0126) and the topiramate and *Lactobacillus johnsonii* treatment groups (*p* = 0.0429).

As butyrate was increased in the ceca of mice treated with topiramate and *Lactobacillus johnsonii*, we performed a simple linear regression of the percentage of butyrate and the abundance of *Lachnospiraceae* and *Ruminococcaeae*, well-known butyrate producers. We found that both families, *Lachnospiraceae* (*p* = 0.0211, *R*^2^ = 0.1951; Figure 5G) and *Ruminococcaeae* (*p* = 0.0008, *R*^2^ = 0.3655; Figure 5H), were positively correlated with the percent of butyrate found in the mice.

### 3.6. Co-administration of topiramate and *Lactobacillus johnsonii* alters the GABA/glutamate ratio in the cortex

**Figure 6:**
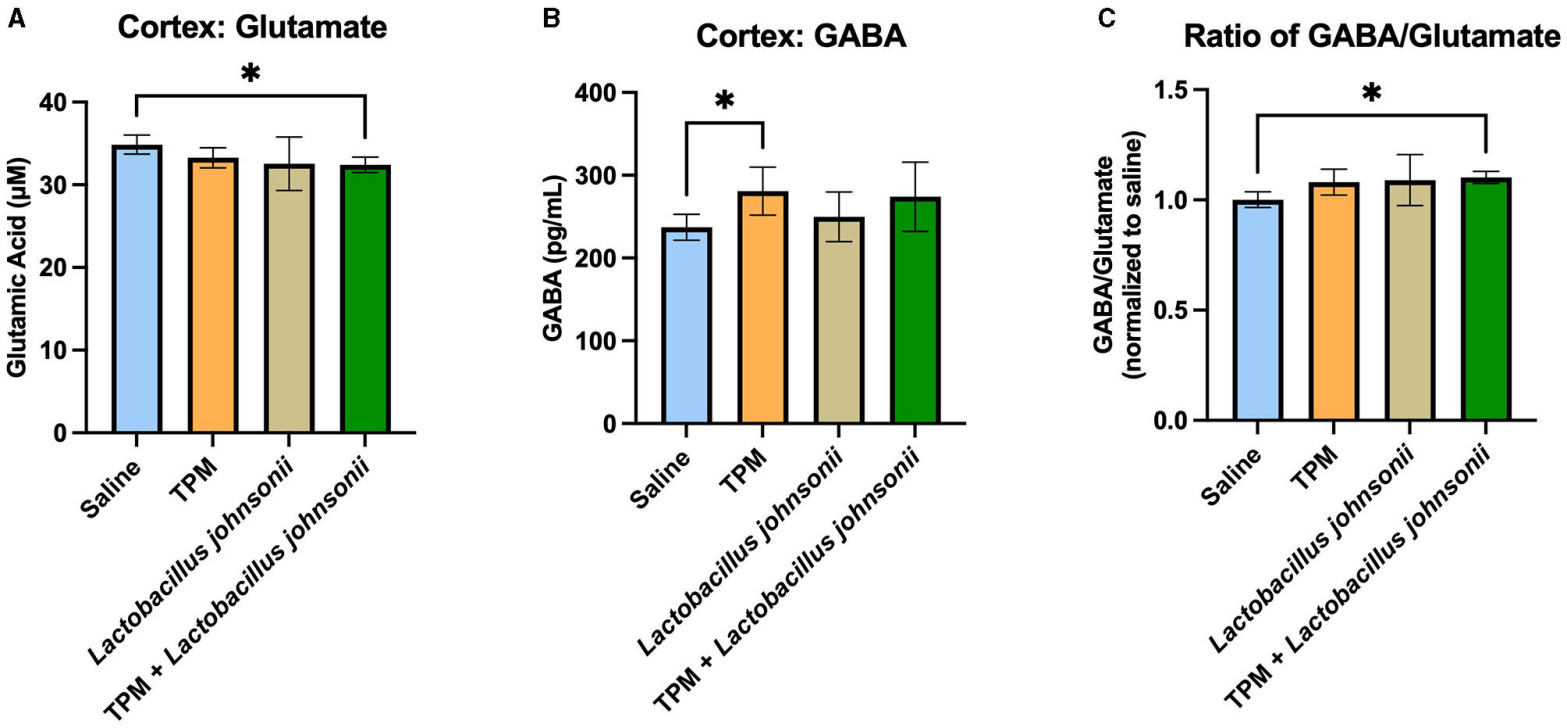
GABA/glutamate ratio is increased due to the co-treatment of topiramate and *Lactobacillus johnsonii*. **(A)** Glutamate was significantly decreased in the cortex of mice who received topiramate and *Lactobacillus johnsonii* together. **(B)** Topiramate treatment alone significantly increased GABA in the cortex of mice. **(C)** The ratio of GABA/glutamate is increased only in the co-treatment of topiramate and *Lactobacillus johnsonii*. Data are shown as mean ± SD. *p < 0.05. Saline = 6, TPM = 7, *Lactobacillus johnsonii* = 7, TPM + *Lactobacillus johnsonii* = 7.

To further determine the mechanisms involved in the reduction of seizure susceptibility due to the co-treatment of topiramate and *Lactobacillus johnsonii*, we evaluated the levels of GABA and glutamate among the groups. The cortex of mice that underwent PTZ-kindling was analyzed for GABA and glutamate concentration. Kruskal-Wallis with Dunn's multiple comparisons test showed a significant decrease in glutamate in the cortex of mice treated with topiramate and *Lactobacillus johnsonii* together when compared with controls (*p* = 0.0495; Figure 6A), but not in topiramate (*p* = 0.6071), or *Lactobacillus johnsonii* treatment alone (0.0943). We also saw an increase in GABA levels in mice that were treated with topiramate alone when compared to saline controls (*p* = 0.0388; Figure 6B), but not in the co-treatment group (*p* = 0.0902). However, there was a significant increase in the GABA/glutamate ratio in the co-treatment group (*p* = 0.0223; Figure 6C), but not in topiramate (*p* = 0.1579) or *Lactobacillus johnsonii* treatment alone (*p* = 0.0862).

## 4. Discussion

The gut microbiome plays a role in reducing seizure susceptibility; however, less is known of ASMs interaction with the gut microbiome *in vivo*. Here, we show that ingestion of topiramate increased the abundance of *Lactobacillus johnsonii*. In addition, only co-treatment of topiramate and *Lactobacillus johnsonii* reduced seizure susceptibility in a PTZ-kindling mouse model compared with topiramate or *Lactobacillus johnsonii* treatment alone. This may in part be attributed to the effect of co-treatment remodeling the gut microbiome to increase butyrate-producing families, such as *Lachnospiraceae*, which in turn increased butyric acid. Importantly, we also accredit the decrease in seizure susceptibility to the reduction in glutamate levels in response to the co-treatment of topiramate and *Lactobacillus johnsonii*.

In our study, we found that topiramate ingestion through drinking water increases *Lactobacillus johnsonii*. It is possible that topiramate's unique structure may contribute to its gut microbiome-altering effect. In addition, topiramate's weak carbonic anhydrase inhibitory activity has been thought to lead to its side effects of metabolic acidosis (Mirza et al., 2009). *Lactobacillus* species can often survive in low pH environments, even as low as a pH of 1, potentially leading to the selection of this naturally occurring species in the mouse gut (Aiba et al., 2015; Davoren et al., 2018). It is also unknown if *Lactobacillus johnsonii* can use the sulfamate-substituted monosaccharide as a carbon source, contributing to its growth. This would be unlikely to affect the host as 70% of topiramate is excreted in the urine and remains unchanged, alluding to an abundance of topiramate in the system (Johnson and Quick, 2023).

*Lactobacillus johnsonii* has been shown to be a beneficial species despite being understudied when compared to its lactobacilli counterparts. Research has shown that *Lactobacillus johnsonii* has immunomodulatory effects in which it can aid immune-checkpoint blockade therapy in colorectal cancer tumor reduction, as well as increase neurotrophic factors in the brain and increase tight-junction proteins at both the intestinal and blood-brain barrier (Mager et al., 2020; Wang et al., 2020; Bai et al., 2022a). In our study, we found that although *Lactobacillus johnsonii* administration alone is not sufficient to reduce seizure susceptibility, in combination with topiramate, *Lactobacillus johnsonii* reduced seizure susceptibility in a PTZ-kindling model. One potential contributing factor to this protective effect is the alteration of the gut microbiome, which increased butyrate, as well as the butyrate-producing bacteria, *Lachnospiraceae*. Of interest, Zhong et al. (2023) recently showed that *Lactobacillus johnsonii* alleviated the development of acute myocardial infarction. They showed that administration of *Lactobacillus johnsonii* increased butyrate, but this effect was mitigated with prior treatment with antibiotics. This result, coupled with our findings, suggests that the administration of *Lactobacillus johnsonii* may alter the composition of the gut microbiome to favor butyrate production and ultimately aid in reduced seizure susceptibility.

We also found a weak, positive correlation between the presence of two well-known butyrate producers, *Lachnospiraceae* and *Ruminococcaceae*, and the percentage of butyrate in the ceca of mice. *Lachnospiraceae* contain metabolic pathways to use lactate, a product *Lactobacillus johnsonii* is capable of producing, to produce butyrate (Vacca et al., 2020; Vazquez-Munoz et al., 2022). It has been documented that butyrate-producing bacteria from the family *Lachnospiraceae* can produce butyrate in the presence of lactate and are even favored in low pH environments (Duncan et al., 2004; Belenguer et al., 2007). Butyrate is one of the most abundant SCFAs in the gut microbiota and is associated with many beneficial effects, ranging from an energy source for colonocytes to neuroprotection in the brain (Roediger, 1982; Sun et al., 2015; Jaworska et al., 2017). Butyrate is also a noted histone-deacetylase (HDAC) inhibitor that can regulate gene expression (Candido et al., 1978). Chronic administration of sodium butyrate reduced seizure susceptibility in a PTZ-kindling and an electrical kindling mouse model, due to the reduction of mitochondrial dysfunction and oxidative stress in the former (Reddy et al., 2018; Li et al., 2021).

A balance between excitatory and inhibitory signaling is imperative in seizure management. Topiramate impacts this balance by enhancing GABA_A_ receptor activity, and it has been shown to increase GABA in the brains of patients (Petroff et al., 2001). In the topiramate-only treatment group, we observed a significant increase in GABA levels in the cortex, as well as a trending increase in the topiramate and *Lactobacillus johnsonii* co-treatment groups. Glutamate reduction only occurred in the co-treatment group but was trending in the *Lactobacillus johnsonii* group. It is possible that the increase in GABA may be driven by topiramate while the decrease in glutamate may be due to the increased presence of *Lactobacillus johnsonii*, and together these changes resulted in a significant increase in the GABA/glutamate ratio only seen in the co-treatment group. Further research needs to elucidate the role of *Lactobacillus johnsonii*'s effect on glutamate levels in the brain, as other research has shown that gut microbiota are capable of altering the GABA/glutamate ratio in the hippocampus of mice, as well as impacting glutamine, a precursor to glutamate, levels in the brain of mice (Kawase et al., 2017; Olson et al., 2018).

To the best of our knowledge, this study is one of the first to show the ability of an ASM to alter the gut microbiome to aid in its anti-seizure effect. This study also highlights the potential for probiotics, such as *Lactobacillus johnsonii*, as an easily applicable adjuvant seizure therapy.

## Data availability statement

The datasets presented in this study can be found in online repositories. The names of the repository/repositories and accession number(s) can be found below: NCBI—PRJNA1004294.

## Ethics statement

The animal study was approved by Virginia Tech Institutional Animal Care and Use Committee. The study was conducted in accordance with the local legislation and institutional requirements.

## Author contributions

KT: conceptualization (equal), investigation (lead), writing—original draft (lead), and writing—review and editing (supporting). MT: investigation (supporting). TF: investigation (supporting), writing—original draft (supporting), and writing—review and editing (supporting). EA: investigation (supporting). SC: conceptualization (equal), writing—review and editing (lead), funding acquisition, and supervision. All authors contributed to the article and approved the submitted version.
