# Supplementary material for: Topiramate alters the gut microbiome to aid in its anti-seizure effect
Source: Front Microbiol. 2023 Oct 10;14:1242856. doi: 10.3389/fmicb.2023.1242856 (PMC10629356; doi:10.3389/fmicb.2023.1242856)
Supplement: Supplementary file 1 [file Data_Sheet_1.PDF]

frontiers

Supplementary Material

Topiramate alters the gut microbiome to aid in its anti-seizure effect

K'Ehleyr Thai, Michael W. Taylor, Tatiane Fernandes, Eunice A. Akinade, Susan L. Campbell*

*** Correspondence:** Susan L. Campbell, PhD: susanc08@vt.edu

1 Supplementary Figures and Tables

1.1 Supplementary Figures

Supplemental Figures

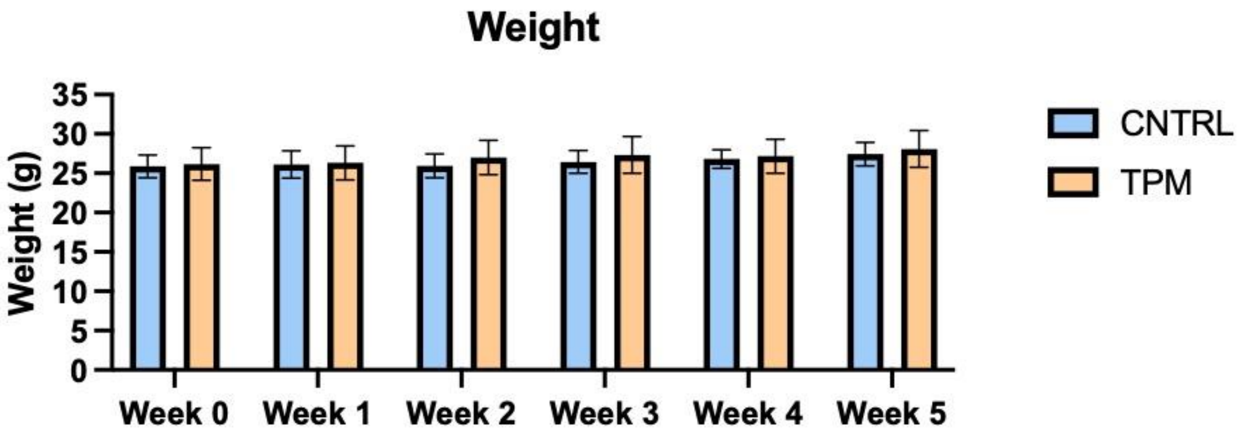

Figure S1. Weight of C57BL/6J naïve mice receiving regular or topiramate-treated drinking water. A repeated measures two-way ANOVA using Geisser-Greenhouse correction showed no significant differences between time and treatment (p=0.4969) or treatment (p=0.5438). There were significant differences over time (p=0.0007) and subject (p<0.0001). Data are shown as mean ±SD. CNTRL=8, TPM=6

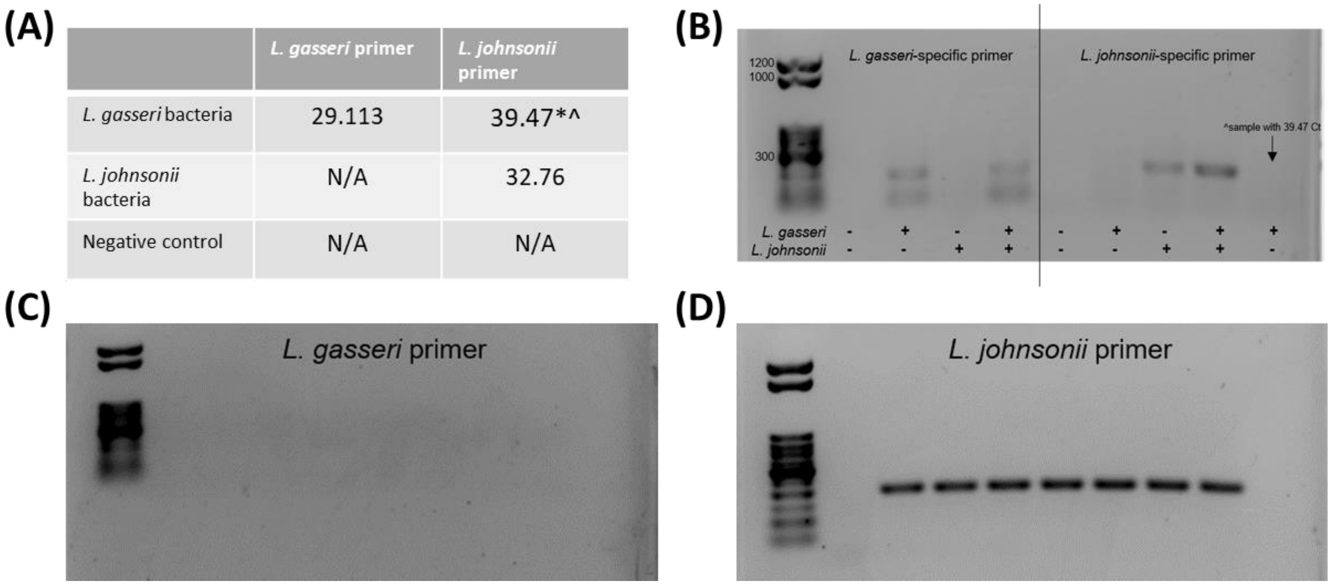

(A)

	L. gasseri primer	*L. johnsonii* primer
L. gasseri bacteria	29.113	39.47*^
L. johnsonii bacteria	N/A	32.76
Negative control	N/A	N/A

Figure S2. *Lactobacillus johnsonii* primer validation and confirmation of presence in fecal samples. (A) Average Ct of triplicates of qPCR samples of bacteria with specified primers. *Lactobacillus gasseri* primer has one out of three triplicates amplify at 39.47. (B) 2% DNA gel of *Lactobacillus gasseri* and *Lactobacillus johnsonii* products after qPCR with known bacterial samples showing that primers are specific to each species. Last lane is the product from the well containing *Lactobacillus gasseri* but using the *Lactobacillus johnsonii* primer, showing no product. (C) Product ran on 2% gel from *Lactobacillus gasseri* primer used on fecal samples of mice from Figure 2 showing no product. (D) Product ran on 2% gel from *Lactobacillus johnsonii* primer used on fecal samples of mice from Figure 2, suggesting *Lactobacillus johnsonii* is in the samples and not *Lactobacillus gasseri* as identified by QIIME2 analysis. *Lactobacillus gasseri* expected product size: 176 bp, *Lactobacillus johnsonii* expected product size: 195 bp.

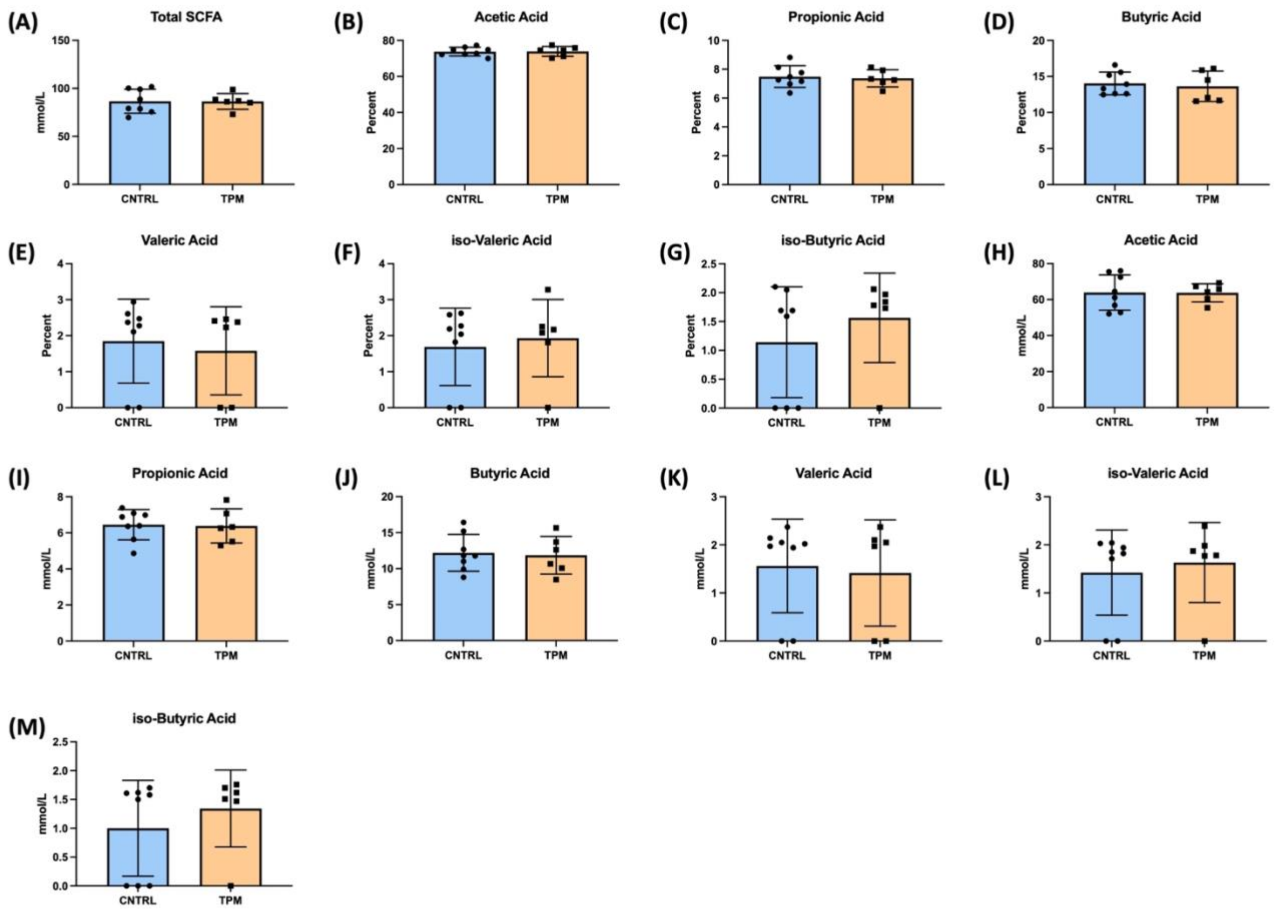

Figure S3. SCFA composition of the ceca of control or topiramate-treated mice. No significant differences were found in (A) total amount of SCFAs, SCFA percentage (B-G), or SCFA concentration (H-M). Data are shown as mean ±SD. CNTRL=8, TPM=6

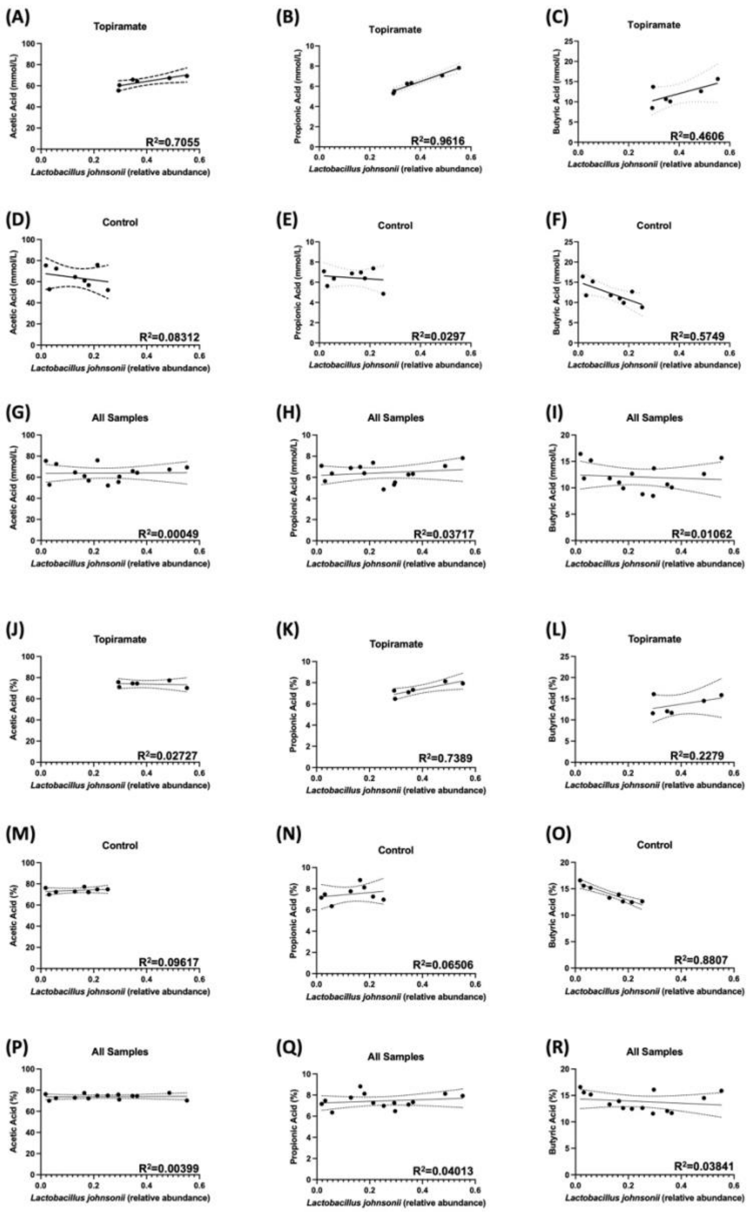

Figure S4. Abundance of *Lactobacillus johnsonii* is positively correlated with the major SCFAs, acetic acid, propionic acid, butyric acid, in topiramate treated, but not control, mice. Concentration of SCFA with relative abundance of *Lactobacillus johnsonii* are shown (A-I). Percentage of SCFA with relative abundance of *Lactobacillus johnsonii* are shown (J-R). Data is shown with regression line and 95% confidence bands. CNTRL=8, TPM=6, ALL=14

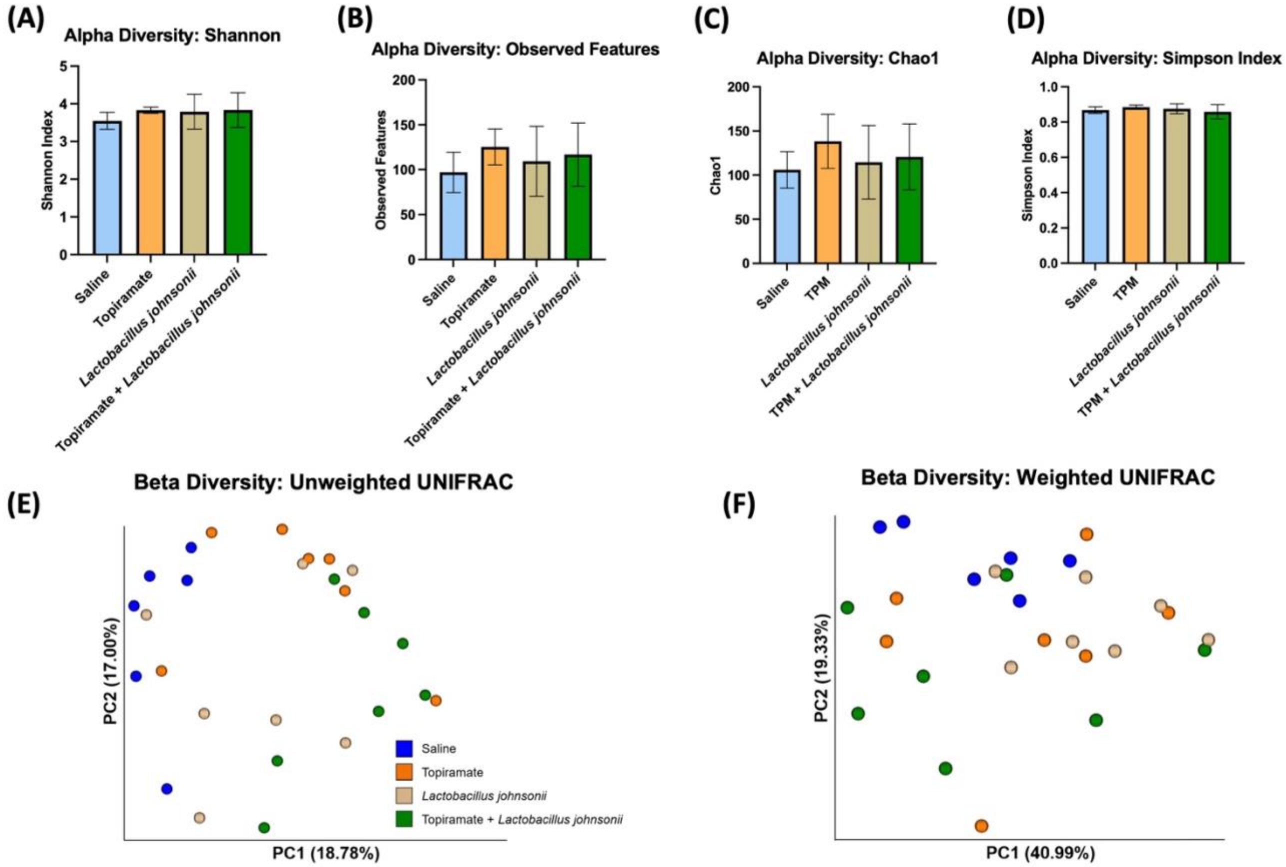

Figure S5. Alpha and beta diversity metrics of the gut microbiome of mice receiving treatment and undergoing PTZ kindling. (A-D) There were no significant differences found in the alpha diversity, Shannon Index, Observed Features, Chao1, or Simpson Index amongst the different treatment groups after PTZ kindling. (E, F) Beta diversity was found to be significantly different in unweighted and weighted UNIFRAC statistical tests when comparing the different treatment groups. Data are shown as mean ±SD. Saline=6, TPM=7, *Lactobacillus johnsonii*=7, TPM + *Lactobacillus johnsonii*=7.

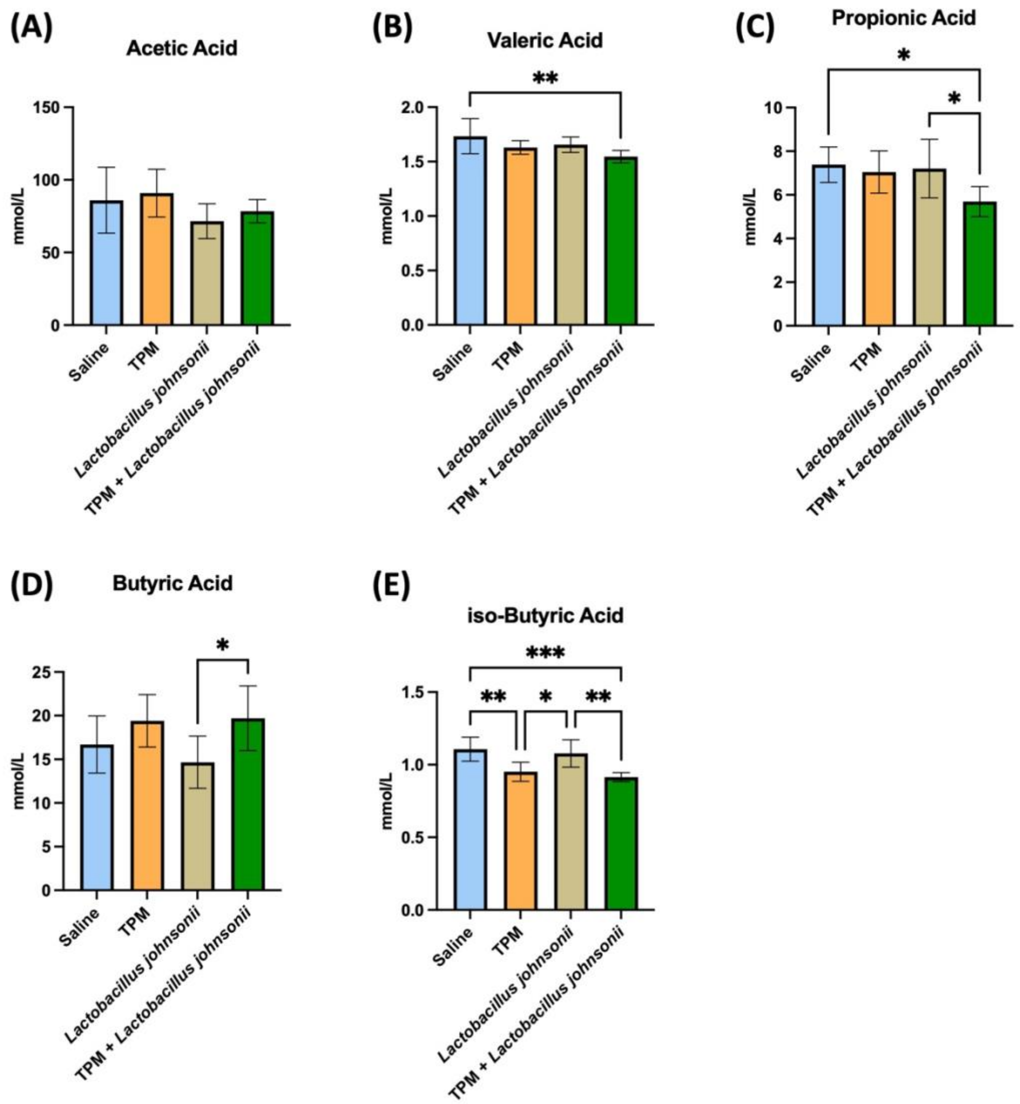

Figure S6. Concentrations of SCFAs amongst different treatment groups that underwent PTZ-kindling. Data are shown as mean ±SD. * $p < 0.05$, ** $p < 0.01$, *** $p < 0.001$. Saline=6, TPM=7, *Lactobacillus johnsonii*=7, TPM + *Lactobacillus johnsonii*=7.
